# Treating Popliteal Fossa Perforating Vein Varicosis with Endovenous Laser Ablation: A Single-Center Observational Study

**DOI:** 10.3390/jcm14103524

**Published:** 2025-05-18

**Authors:** Lars Müller, Isabel Schmitz-Rode, Bachar el Jamal, Syrus Karsai, Eike Sebastian Debus

**Affiliations:** 1 Department of Vascular Surgery, Dermatologikum Hamburg GmbH, 20354 Hamburg, Germany; isabel.schmitz@dermatologikum.de (I.S.-R.); bachar.eljamal@dermatologikum.de (B.e.J.); 2 Department of Vascular Medicine, University Heart and Vascular Center, University Medical Center Hamburg-Eppendorf, 20251 Hamburg, Germany; s.debus@uke.de; 3 Department of Dermatology, Dermatologikum Hamburg GmbH, 20354 Hamburg, Germany; s.karsai@dermatologikum.de; 4 Department of Dermatology, University Medical Center Hamburg-Eppendorf, 20251 Hamburg, Germany

**Keywords:** chronic venous insufficiency, great saphenous vein, GSV, small saphenous vein, SSV, laser ablation, EVLA, perforator, varicose veins

## Abstract

**Background:** Treating varicosities originating from a popliteal fossa perforating vein (PFPV) is challenging due to their proximity to nerves and complex morphology. Data on endovenous laser ablation (EVLA) for PFPV varicosis are limited. **Methods:** This retrospective, single-center study reviewed all primary varicose vein surgeries from May 2021 to December 2024. Only primary PFPV varicosis cases with CEAP stage C2s or higher were included. Patients with recurrent disease or primary truncal insufficiency due to reflux from the saphenopopliteal junction were excluded. EVLA was performed using 1470 nm radial laser catheters, targeting the reflux source and downstream varicose segments. Tumescent solution was applied to protect the surrounding structures. The primary outcome was early technical success via duplex ultrasound; the secondary outcome was the complication rate. **Results:** Of the 2375 limbs treated, 44 (1.9%) involved PFPV. The cohort included 16 men (36%) and 28 women (64%), with a mean age of 54. The median follow-up was 14 days. Technical success was achieved in 41 cases (93.2%). Foam sclerotherapy with polidocanol was performed in eight patients (18.2%), exclusively for superficial residual varicosities and never simultaneously with EVLA. Three treatment failures required re-operation, two of which were successfully re-treated. Minor postoperative complications occurred in two patients (4.5%). No nerve injuries or thrombotic events were observed. **Conclusions:** EVLA shows promising very early technical efficacy, with low morbidity, for treating PFPV varicosis. Based on our findings, prospective studies investigating the mid- and long-term outcomes of this technique are warranted to further validate its clinical utility.

## 1. Introduction

Reflux through a popliteal fossa perforating vein (PFPV) is a recognized cause of varicose vein disease. PFPV, also designated as the perforator of the popliteal fossa [1,2], popliteal fossa vein [3,4] and historically referred to as the Thiery vein [3,5,6], originates from the deep venous system within the popliteal fossa, with reflux points independent of both the great saphenous vein (GSV) and the small saphenous vein (SSV) [7]. PFPV varicosis typically manifests as tortuous, meandering, and large-caliber venous segments beneath and above the popliteal fascia. The veins often connect to symptomatic subcutaneous varicosities located near the flexor crease behind the knee [2,4,5]. Additionally, the PFPV sometimes serves as a source of reflux in cases of segmental insufficiency of the SSV, even when the sapheno–popliteal junction (SPJ) remains competent [1].

The prevalence of PFPV reflux in patients with varicosis has been reported to range from 0.84% [7] to 7.06% [8]. PFPV varicosis is strongly associated with other forms of superficial venous reflux, particularly truncal venous insufficiency [4]. In Delis’s study, patients with PFPV exhibited more severe symptoms, as evidenced by the higher venous clinical severity scores (VCSSs) compared with those with other types of venous reflux [4]. Additional reports highlight the significant negative hemodynamic consequences of PFPV-related reflux, including chronic venous insufficiency (CVI) with dilatation of the deep venous system [7,9].

Contemporary practice guidelines define the treatment indication for perforating vein incompetence when it is responsible for clinically relevant varicose veins [10]. Both foam sclerotherapy and endovenous thermal procedures are recommended treatment options [10,11]. However, specific evidence-based guidelines for treating PFPVs are lacking, and the literature primarily consists of observational studies outlining various treatment options, including surgical resection [12], endoscopically assisted resection [13], and endovenous laser ablation (EVLA) [3,9].

Thermal ablation procedures are more challenging than the primary thermal ablation of straight truncal vein segments due to the unique morphology and anatomical location of the PFPV in the popliteal fossa, near nervous structures. However, studies suggest that thermal procedures offer more effective closure than foam sclerotherapy, which is a widely used method [10,14]. Recent advancements in ablation techniques and medical devices have made it possible to effectively treat shorter or even meandering vein segments.

This observational study aimed to evaluate the experience of a single center with thermal ablation using radial laser energy at 1470 nm. Specifically, this study focused on the outcome in patients with varicose veins secondary to PFPV. The chosen approach was to first assess the technical feasibility and initial ablation success using routine data collected during patient treatment.

## 2. Materials and Methods

### 2.1. Study Design

This retrospective, single-center, single-operator study reviewed all venous surgeries conducted over 44 months, from May 2021 to December 2024. All cases involving the treatment of PFPV reflux were included in the study cohort. Data were retrieved from the clinic information system and collected in a fully anonymized form, ensuring that the later identification of participants was not possible. Upon request, the Ethics Committee of the Hamburg Medical Association waived the requirement for consultation due to the retrospective study design and the exclusive use of fully anonymized data (reference no: PV7252-4650_2-WF).

### 2.2. Preoperative Diagnostics

In addition to collecting medical histories and performing a physical examination, including inspection and palpation of the affected legs, the clinical stage was determined using the clinical, etiological, anatomic, and pathophysiological (CEAP) classification. Only cases with stage C2 with symptoms (C2s) or higher were eligible for intervention with thermal ablation. A standardized duplex ultrasound examination [15] was then performed with the patient standing. This assessment examined both the superficial and deep venous systems using a linear probe and a duplex ultrasound device (Logiq p7, GE Healthcare, Chicago, IL, USA). All patients with a clearly identifiable PFPV (>4 mm diameter) with reflux (>0.5 s) were included. Patients who had a previous surgical or interventional treatment of the SSV in the same leg were excluded. Additionally, cases where the varicosity was caused by reflux from the proximal SSV through the saphenopopliteal junction were excluded. However, cases involving segmental, distal insufficiency of the SSV trunk, filled by branches of a popliteal perforator vein, were included.

### 2.3. Interventional Treatment

**Figure 1:**
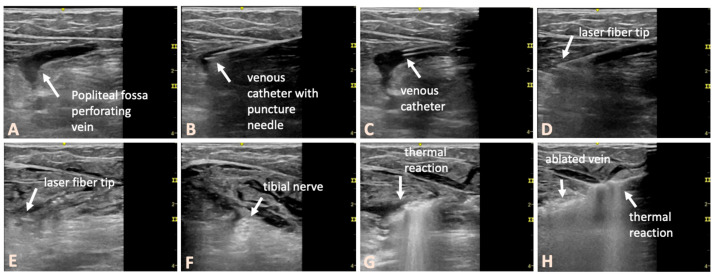
Representative intraoperative ultrasound images illustrating the treatment procedure using the example of a left-sided PFPV in a female patient with non-complex anatomy. (**A**) The transducer is positioned in a longitudinal, in-plane orientation, showing the perforating vein and its course beneath the popliteal fascia. (**B**) The vein is punctured with the indwelling venous cannula. (**C**) The hollow needle is then removed. (**D**) The laser fiber, visible via the slight acoustic shadow in the first cm, is positioned, and the venous catheter is withdrawn. (**E**) The tumescent solution is then perivenously injected. (**F**) The nerve structures of the popliteal fossa are only visible in the transverse orientation. Care must be taken to ensure that there is sufficient distance between the tip of the intravenous catheter and the existing nerve structures. If necessary, a targeted injection of tumescent solution is performed to increase the distance. (**G**,**H**) The laser energy is applied under continuous sonographic monitoring, even during the continuous withdrawal of the catheter.

After obtaining informed consent, the surgeries were performed in a dedicated operating room under strict sterile conditions, with the patient in the prone position. Throughout the procedure, continuous ultrasound visualization was used (Logiq e, GE Healthcare, Chicago, IL, USA). Figure 1 shows an illustrative example of the treatment procedure using intraoperative ultrasound images. Conventional 16 GA venous cannulas were employed to access the targeted veins. The PFPV was punctured in such a way that the placement of the laser catheter enabled the ablation of the longest vein segment, with the catheter tip positioned as close as possible to the junction with the deep vein system. In complex cases, multiple cannulas were used, which were placed preparatively into the veins to be ablated. This step ensured secure venous access before the perivenous tumescent anesthesia was administered. After the placement of the venous cannulas, we inserted the laser catheter (1470 nm ELVeS Radial 2ring slim, Biolitec AG, Vienna, Austria) into the most proximally located venous segment. A 4 °C tumescent solution (1000 mL of physiological saline + 50 mL of 1% mepivacaine + 8 mL of 8.4% sodium bicarbonate) was then perivenously infiltrated. Particular attention was paid to creating a fluid sleeve around the treated veins to protect the surrounding tissues. The anatomical relationships with nerve structures in the popliteal fossa, including the tibial nerve, medial sural cutaneous nerve, and common peroneal nerve, were considered (Figure 1). The nerves could be well visualized in the transverse ultrasound view and, if necessary, displaced using targeted tumescent injection.

The ablation was performed at a power setting of 8 W. The linear energy density (LEED) was calculated to be approximately 10 J per mm of vein diameter per cm of treated vein, depending on the diameter of the veins being treated. No simultaneous foam sclerotherapy with polidocanol was performed during the laser treatment sessions. At the patient’s request, the treatment could be conducted under mild sedation with propofol, supervised by an anesthesiologist. Postoperatively, sterile adhesive bandages with eccentric compression were applied to the puncture sites, but no circular compression bandages or compression stockings were used. All patients received a single intraoperative dose of low-molecular-weight heparin as a prophylactic measure. Motor nerve function was assessed immediately following the procedure.

### 2.4. Postoperative Follow-Up

**Figure 2:**
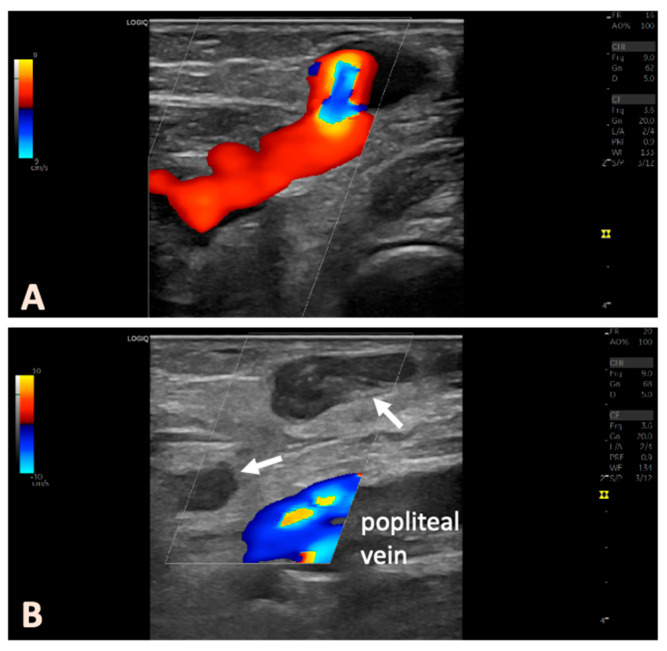
Pre- and post-interventional duplex ultrasound in an example of a patient with a right-sided PFPV. The ultrasound probe is positioned in a longitudinal orientation. (**A**) preoperative findings; (**B**) postinterventional findings, with the thermally treated veins below and above the popliteal fascia occluded (arrows).

A follow-up appointment was scheduled within the first 4 weeks post intervention, preferably between 10 and 14 days. This follow-up included both clinical and duplex ultrasound examinations. We assessed for any neural deficits, and duplex ultrasound was used to confirm that the deep venous system was patent, with no evidence of thrombosis or endovenous heat-induced thrombosis (EHIT). Morbidity was defined as any condition requiring interventional or pharmacological treatment and necessitating further clinical follow-up. Accordingly, transient symptoms such as minor hematomas or localized, phlebitic induration of superficial veins were not classified as complications. Treatment success—defined as thermal closure of the proximal, subfascial portion of the PFPV, with the absence of venous flow and no prolonged reflux in the associated varicosities—was documented using duplex ultrasonography (Figure 2). In our routine clinical practice, venous clinical severity scores (VCSSs) were not recorded before or after the intervention; treatment success was evaluated exclusively based on ultrasonographic findings. In some cases, foam sclerotherapy was performed for aesthetically disturbing superficial varices, or additional sessions were scheduled with the patients if necessary. Patients were always offered further follow-up examinations. It was also strongly recommended to seek our help in case of unclear complaints or persistent or new venous symptoms.

### 2.5. Data Analysis and Statistics

The primary outcome parameter was the success rate of treatment, determined at the postoperative follow-up examination. The secondary outcome parameter was the morbidity related to the ablation procedure. Additionally, results from subsequent follow-up examinations were evaluated when available. Descriptive statistics were used for data analysis, including means with standard deviations for normally distributed data, medians with ranges for non-normally distributed data, and frequencies for categorical data.

## 3. Results

Primary interventions for varicose veins were performed on 2375 limbs during the study period. Of these, 44 treatments (1.9%) were conducted due to the diagnosis of a PFPV (Table 1). This cohort consisted of 16 men and 28 women, with a mean age of 54 ± 15.2 years. Most patients (84.1%) presented with symptomatic varicose veins at clinical stage C2; seven patients (15.9%) had stage C3. Preoperative diagnostics revealed additional, treatable reflux in other venous segments in 20 patients (45.5%). However, cases involving primary truncal venous insufficiency in the SSV territory due to reflux from the SPJ on the same side were excluded from this study. In 13 individuals (29.5%), there was GSV incompetence on the contralateral side, and 1 patient (2.3%) presented with truncal varicosities of the SSV on the opposite side. A total of six patients (13.6%) had truncal venous insufficiency in the GSV territory in the same limb, and one patient (2.3%) had bilateral GSV insufficiency. The average maximal diameter of the varicosity associated with the perforator vein, located below the popliteal fascia, measured 7.0 ± 2.2 mm while standing.

In all cases, intraoperative puncture, cannulation, and ablation were considered successful. No additional sclerotherapy with polidocanol foam was performed during the EVLA session. In 17 patients (38.6%), adjunctive procedures using EVLA were carried out on other venous segments in the same session, excluding cases with small saphenous vein insufficiency on the same side. In nine cases (20.5%), patients opted for sedation with propofol during the procedure. Tumescent anesthesia was used in all cases, primarily to create a thermal barrier between the nerve structures in the popliteal fossa and the veins, as well as between superficial veins and the skin. Careful visualization of the nerves was ensured to assess any close anatomical relationships with the veins. The sciatic, common peroneal, tibial, and medial sural cutaneous nerves could be traced using cross-sectional ultrasound. In cases of close proximity or contact between the laser catheter and a nerve, the targeted injection of tumescent solution typically displaced the nerve safely. The morphology of the findings varied. In some cases, treating a short proximal vein segment was sufficient, while in others, complex and twisted varices were found below the popliteal fascia, with additional dependent varices above the fascia. As a result, there was a broad range of treated venous lengths and total applied energies (Table 1).

The recommended follow-up examination was attended by all patients (100% follow-up rate). The median time period of this follow-up examination was 14 days (interquartile range: 10–26 days). The duplex ultrasound examination showed complete technical ablation success in 41 out of 44 cases (93.2%). In eight patients (18.2%), sclerotherapy with polidocanol foam was performed for superficial, dependent varices, although the inflow in the popliteal fossa was appropriately closed with laser treatment. In the three patients (6.8%) with an incompletely closed inflow perforator vein, a repeat EVLA treatment was offered. These insufficient closures closely resembled the initial findings and were, therefore, classified as treatment failures. It is assumed that the underlying cause was inadequate thermal energy delivery to the vein wall or a paravasal positioning of the catheter tip, which went undetected during the procedure. Two patients opted for the re-treatment, and in both cases, the redo procedure was fully successful. One patient initially declined further intervention.

Severe complications, such as persistent neurological deficits, thrombosis, or EHIT, were not observed. Mild, transient symptoms, including increased local pain, occurred in two cases (4.5%), which were successfully managed with analgesics. Postoperative symptoms that did not require medication, interventional measures, or additional follow-up visits were not documented.

In the further course after treatment, only a few patients presented again within one year and could be re-examined (see Table 2). All patients were explicitly instructed to present in the event of new or persistent venous complaints. No additional recurrences were observed during this period. In one patient with a regular ablation result in the postoperative control, and without further follow-up examinations in the first year, a recurrence requiring treatment occurred after 15 months. One patient was diagnosed with a recurrence 34 months postoperatively, which was not present at the previous follow-up 18 months after treatment. Both patients were treated with a repeat EVLA, and the first follow-up showed that the procedures were technically successful.

## 4. Discussion

PFPVs typically lack anatomical or topographical connections to the truncal veins at the site of reflux, classifying them as non-saphenous reflux. Two published observational studies involving 835 and 2036 limbs with signs of CVI reported non-saphenous reflux in approximately 10% [7] and 8% [16] of cases, respectively. PFPVs, a subset of this reflux type, were present in 0.84% [7] and 1.03% [16] of cases. The 1.9% prevalence of superficial reflux from a PFPV observed in our cohort is consistent with these rates, as well as others [2,3,7,8,16]. However, patients with superficial popliteal varices, which may include varicosities from smaller PFPVs, often opt for sclerotherapy first, particularly in cases with minor manifestations and small vein diameters. As a result, our study may not fully capture the true incidence of this type of reflux. In our study of 44 consecutive limbs, 84.1% of cases were in the symptomatic C2 stage, although other venous areas were affected in 45.5% of extremities. While these cases represented less severe forms of CVI, without skin changes or ulcerations (CEAP C4–C6), patients still experienced significant discomfort and aesthetic impairment, which prompted the need for invasive treatment.

Our cohort study showed that EVLA for PFPV achieves acceptable initial success rates with low perioperative morbidity. However, comparative data on the efficacy of EVLA for treating this form of superficial reflux are limited. In a 2015 study, Parikov and colleagues reported on a cohort of 40 patients treated with radial-emitting laser fibers at 1470 nm, achieving a success rate of 97.5%, with mild paresthesia occurring in 2.5% of cases [3]. To the best of our knowledge, no published study has yet reproduced these results. In our study, we observed a success rate of 93.2% in 44 patients, with no paresthesia and similarly low morbidity. These results, combined with increasing clinical experience, suggest that EVLA is an effective and justified treatment for PFPV. However, this statement can currently only be applied to the very early success of technical treatment, and further data on the mid- and long-term outcomes are needed. Alternative surgical treatments for popliteal perforators include open resection [12] or endoscopic-assisted resection [13], both of which have shown no recurrences, although complications, such as wound infections, were observed in a small percentage of cases.

We included only cases where the vein size, reflux extent, and vein location made thermal ablation a safer and more effective choice compared with foam sclerotherapy. The results of thermal treatment for perforating veins in the literature, ranging from 66% to 95.6% [14,17,18], do not match the outcomes seen with the thermal treatment of truncal veins. However, when directly compared, perforating vein treatment outcomes are slightly better than those of foam sclerotherapy [10,14]. While sclerotherapy is effective, particularly for superficial varicose veins, the risk of deep vein occlusion or thrombosis from excessive sclerosant has raised concerns, making thermal ablation of the proximal reflux point the preferred approach. For this reason, we avoided simultaneous sclerotherapy with EVLA, despite positive reports of its use. A recent observational study involving a large cohort found that combining EVLA with foam sclerotherapy carries a sufficiently low complication rate, particularly regarding thrombotic complications [19], suggesting this approach may be suitable for more complex PFPV cases.

The 1470 nm wavelength has been widely used to date for laser ablation in varicose vein disease. Other thermal devices, including radiofrequency catheters, may also potentially be suitable for these treatments. Selective heat application limited to the vein wall would be advantageous to minimize potential thermal damage to anatomical structures. Current reports suggest that longer wavelengths, due to their higher laser energy absorption properties in water, allow for more precise restriction of the heat zone to the vein wall, thereby avoiding damage to the surrounding tissue. This may potentially result in less pain and paresthesia [20,21,22]. Therefore, it might be advisable to consider using the 1940 nm wavelength for safety reasons or to assess its benefits, particularly given the proximity of nerves in the popliteal fossa.

The direct puncture technique, which is used to access varicose veins deeper beneath the popliteal fascia, is similar to the methods employed for treating recurrent varices after small saphenous vein surgeries [23]. Since recurrence in small saphenous veins can mimic primary perforating vein pathologies, we excluded patients with prior small saphenous vein treatments [24]. Compared to primary trunk vein insufficiencies, treating perforating veins is technically more challenging. Clinical guidelines indicate that these conditions may not always be ideal candidates for thermal treatments [10]. The management becomes even more complex when large varices are connected to the PFPV. Whenever possible, we thermally occlude larger varicose vein clusters, using techniques such as multiple punctures or the hedgehog method [25,26]. Successful cannulation requires proficiency in both cross-sectional and longitudinal ultrasound views. For a complex morphology with multiple twisted or angulated vein segments to be treated, it is advisable to prepare several cannulas in advance to ensure effective access before the accumulation of tumescent fluid restricts visibility [23]. We experienced three cases of treatment failure, and two patients were successfully re-treated using the same technique. This decision was based on ultrasound findings, which showed no contraindications to immediately redo thermal treatment. As such, in some cases, short or curved venous segments may appear to be adequately treated intraoperatively but then turn out not to be occluded at follow-up. In these instances, retreatment can be an effective solution.

## 5. Limitations

The generalizability of our findings may be limited due to the retrospective design of this study, which was conducted at a single specialized center. This analysis sought to provide a comprehensive overview of all treatments and initial postoperative outcomes within a defined timeframe. However, it did not offer sufficient insights into the mid- and long-term treatment results. This limitation is partly because multiple routine follow-ups—without symptoms or clinical suspicion after varicose vein treatment—can be a burden for both patients and healthcare providers. A prospective study design would be necessary to obtain more data on treatment efficacy, with efforts focused on minimizing dropout rates. The current analysis may, at least, serve as a foundation for future studies.

## 6. Conclusions

The EVLA of popliteal fossa perforating veins using a 1470 nm laser catheter with radial emission is technically feasible, even in the presence of anatomical challenges. Based on our retrospective data, we can conclude that this offers promising early technical efficacy. Our data can, thus, serve as a basis for prospective studies. Such studies are essential to evaluate the medium- and long-term effectiveness of the treatment. Potentially, the technique can be considered as an alternative to foam sclerotherapy or open surgical interventions. In cases where the initial EVLA is not fully successful, a redo procedure may prove to be an effective solution.

## Figures and Tables

**Table 1:** Baseline characteristics.

**Characteristic**	**Value**
Number of patients	44
Sex; n (%) Male Female	16 (36.4) 28 (63.6)
Age (years); mean (± SD)	54 ± 15.2
Body mass index (kg/m2); mean (± SD)	24.3 ± 3.3 ^a^
Side; n (%) Left Right	26 (59.1) 18 (40.9)
CEAP C2 C3	37 (84.1) 7 (15.9)
Vein diameter (mm); mean (± SD)	7.0 ± 2.2
Energy output (J); median; range, IQR	317; 99–1681; 234–503 ^b^
Treated vein length (cm); median; range, IQR	4; 2–28; 3–7 ^b^
LEED (J/cm); median; range, IQR	67; 27–148; 52–90 ^b^
Sedation Yes No	9 (20.5) 35 (79.5)

SD: standard deviation; CEAP: clinical, etiological, anatomic, and pathophysiological classification; IQR: interquartile range; LEED: average linear endovenous energy density; ^a^ data missing for three patients; ^b^ data missing for three limbs.

**Table 2:** Postoperative treatment results.

Follow-Up Interval	Patients with Available Follow-Up Data	Cumulative Number of Detected Recurrences
Postoperative visit	44/44 (100%)	3
3 months	13/44 (29.5%)	3
6 months	9/44 (20.5%)	3
12 months	4/44 (9.1%)	3

## Data Availability

The original contributions presented in this study are included in this article/the Supplementary Materials. Further inquiries can be directed to the corresponding author.

## Supplementary Materials

The following supporting information can be downloaded at https://www.mdpi.com/article/10.3390/jcm14103524/s1. Table S1: Raw data.

## Abbreviations

The following abbreviations were used in this manuscript:

MDPI	Multidisciplinary Digital Publishing Institute
DOAJ	Directory of Open-Access Journals
TLA	Three-letter acronym
LD	Linear dichroism
